# Sickle Cell Trait Modulates the Proteome and Phosphoproteome of *Plasmodium falciparum*-Infected Erythrocytes

**DOI:** 10.3389/fcimb.2021.637604

**Published:** 2021-03-24

**Authors:** Margaux Chauvet, Cerina Chhuon, Joanna Lipecka, Sébastien Dechavanne, Célia Dechavanne, Murielle Lohezic, Margherita Ortalli, Damien Pineau, Jean-Antoine Ribeil, Sandra Manceau, Caroline Le Van Kim, Adrian J. F. Luty, Florence Migot-Nabias, Slim Azouzi, Ida Chiara Guerrera, Anaïs Merckx

**Affiliations:** ^1^ Université de Paris, MERIT, IRD, Paris, France; ^2^ Laboratoire d’Excellence GR-Ex, Paris, France; ^3^ Université de Paris, Proteomics Platform Necker, Structure Fédérative de Recherche Necker, Inserm US24/CNRS, UMS3633, Paris, France; ^4^ Université de Paris, Inserm, BIGR, Paris, France; ^5^ Institut National de la Transfusion Sanguine, Paris, France; ^6^ Biotherapy Department, Necker Children’s Hospital, Assistance Publique-Hôpitaux de Paris, Paris, France

**Keywords:** *Plasmodium falciparum*, hemoglobin S, erythrocyte, membrane phosphorylation, proteomics

## Abstract

The high prevalence of sickle cell disease in some human populations likely results from the protection afforded against severe *Plasmodium falciparum* malaria and death by heterozygous carriage of HbS. *P. falciparum* remodels the erythrocyte membrane and skeleton, displaying parasite proteins at the erythrocyte surface that interact with key human proteins in the Ankyrin R and 4.1R complexes. Oxidative stress generated by HbS, as well as by parasite invasion, disrupts the kinase/phosphatase balance, potentially interfering with the molecular interactions between human and parasite proteins. HbS is known to be associated with abnormal membrane display of parasite antigens. Studying the proteome and the phosphoproteome of red cell membrane extracts from *P. falciparum* infected and non-infected erythrocytes, we show here that HbS heterozygous carriage, combined with infection, modulates the phosphorylation of erythrocyte membrane transporters and skeletal proteins as well as of parasite proteins. Our results highlight modifications of Ser-/Thr- and/or Tyr- phosphorylation in key human proteins, such as ankyrin, β-adducin, β-spectrin and Band 3, and key parasite proteins, such as RESA or MESA. Altered phosphorylation patterns could disturb the interactions within membrane protein complexes, affect nutrient uptake and the infected erythrocyte cytoadherence phenomenon, thus lessening the severity of malaria symptoms.

## Introduction

Hemoglobin (Hb) S erythrocyte abnormality is due to a genetic mutation resulting from the replacement of a glutamate at the sixth position within the β-globin chain by a valine in normal Hb (HbA)  (Flint et al., 1998). The homozygous carriage of HbS (HbSS) results in sickle cell disease (SCD) which has serious pathological consequences such as anemia and vaso-occlusive crises and can be life-threatening. SCD is highly prevalent in human populations living in malaria endemic areas. The highest frequencies of HbS homozygous and heterozygous forms are indeed observed in sub-Saharan Africa, the Middle East, and India, reaching up to 25%  (Migot-Nabias et al., 2000) and could result from the protection afforded by HbS heterozygous carriage (HbAS) against severe *Plasmodium falciparum* malaria-related symptoms and death without serious hematological disadvantage  (Piel et al., 2010; Gonçalves et al., 2016). The HbAS genotype, commonly called sickle cell trait, is less clinically visible than the HbSS one.

*P. falciparum* asexual multiplication within human red blood cells (RBCs) leads to malaria symptoms. During this phase, *P. falciparum* exports proteins into the erythrocyte cytosol and membrane in order to remodel the host cell and allow it to prosper in this environment. Some of the most profound changes in infected erythrocytes comprise protrusions at the surface, termed « knobs », and vesicular cisternae in the RBC cytosol, called « maurer’s clefts » (MCs). Export of proteins involves the remodeling of host actin by the parasite, to connect MCs and MCs-originated vesicles to the host cell cytoskeleton and membrane  (Rug et al., 2014). Knobs display « *P. falciparum* erythrocyte membrane protein 1 » (*Pf*EMP1) that mediates the cytoadherence of infected RBCs (iRBCs) to microvascular blood vessel endothelium, thus preventing clearance of iRBCs by the spleen. The resulting obstruction of the microvasculature in the brain and other organs is considered to be one of the major contributors to the pathology associated with the most severe forms of malaria  (Miller et al., 2002).

The molecular mechanisms of the protection conferred against severe malaria symptoms by abnormal HbS are still only partially understood and under active investigation. Previous studies showed that HbAS erythrocytes display impaired parasite-induced host actin reorganization and incompletely developed MCs, leading to reduced and impaired protein export  (Cholera et al., 2008; Fairhurst et al., 2012). Furthermore, these RBCs display reduced amounts of adhesins which are aberrantly presented as they are anchored in enlarged and dispersed knobs, correlating with reduced cytoadherence capacity. Oxidative stress caused by HbS instability underlies the aberrant development of the parasite, insofar as an oxidative insult conferred to HbAA RBCs can promote the same phenotypic discrepancies  (Cyrklaff et al., 2016).

The underlying erythrocyte membrane based-skeleton is known to play a major role in the function of the RBC membrane as well as in parasite knobs’ organization. This cytoskeleton is physically linked to the membrane by vertical interactions at two key sites: the AnkyrinR and the 4.1R complexes  (Mankelow et al., 2012). Abnormalities in erythrocyte membrane proteins have been observed in RBC disorders and are often associated with an increase of oxidative stress on proteins themselves or with perturbations in intracellular signaling pathways such as the balance between phosphatase/kinase functions  (Pantaleo et al., 2010a). The phosphoproteome of the AnkyrinR complex and the erythrocyte membrane  (George et al., 2010) is affected in HbSS RBCs. This altered phosphorylation of skeleton proteins may affect membrane deformability, increasing fragility and rigidity of the erythrocyte  (George et al., 2010).

Both parasite infection and HbS promote oxidative stress in RBCs, which can subsequently modify the phosphorylative status of human and parasite proteins. Phosphorylation is indeed a key mechanism for parasite development and could be disturbed by HbS. Previous studies showed that the kinase/phosphatase equilibrium alteration could influence cytoadherence of iRBCs to endothelial receptors  (Dorin-Semblat et al., 2019), erythrocyte membrane channel activities  (Merckx et al., 2008) and membrane mechanical properties of iRBCs  (Nunes et al., 2010). Therefore, we hypothesized that modulation of protein phosphorylation in iRBCs could be one mechanism underlying the relative protection conferred by HbS against malaria.

The present study has investigated the impact of heterozygous HbS carriage, as well as of *P. falciparum* infection, on the phosphorylative states of protein components of the erythrocyte cytoskeleton and membrane, and of parasite proteins. To this end, the phosphoproteome and the proteome of erythrocyte ghosts and parasite proteins from *P. falciparum* infected and non-infected homozygous HbAA and heterozygous HbAS RBCs were analyzed by mass spectrometry after TiO_2_ enrichment, and by western blots.

The mass spectrometry analysis describes phosphorylation differences of both RBCs and parasite proteins. Those alterations concerned erythrocyte skeleton - interacting parasite proteins, erythrocyte transporters, membrane and skeletal proteins and could be involved in the regulation of molecular interactions within membrane protein complexes, such as the cytoadherence complex, and in the regulation of the functions of these proteins.

## Materials and Methods

### Blood Samples

HbAA and HbAS erythrocytes were obtained from voluntary donors, after written informed consent obtained in accordance with the Declaration of Helsinki at Necker‐Enfants‐Malades hospital (Committee for the Protection of Persons n°DC 2014-2272). This study was approved and conducted according to institutional ethical guidelines of the National Institute for Blood Transfusion (INTS, Paris, France). Blood was collected in tubes containing citrate-phosphate-dextrose with adenine (CPDA). RBCs were then separated from plasma and leucocytes by three washings in RPMI 1640 medium (*Gibco-ThermoFisher Scientific*) and were stored maximum 24hrs at 4°C before use, or cryoconserved.

### DNA Extraction

Genomic DNA was extracted from the donor’s leucocytes with the DNeasy Blood and Tissue Kit (*Qiagen)*. The concentration and the quality of DNA were evaluated by measuring the absorbance at 260 nm and 280 nm with a Nanodrop spectrophotometer.

### Hemoglobin Genotyping

Hemoglobin genotypes were determined by polymerase chain reaction – restriction fragments length polymorphisms (PCR-RFLP) adapted from Badaut et al. (2015), and according to the GoTaqFlexi DNA Polymerase (*Promega*) manufacturer requirements.

### *Plasmodium falciparum*-Infected Erythrocyte Culture

*P. falciparum* strain 3D7 was grown *in vitro* in human HbAA and HbAS erythrocytes according to the procedures of “Methods in Malaria Research” (https://www.beiresources.org/portals/2/MR4/Methods_In_Malaria_Research-6th_edition.pdf) adapted from Trager and Jensen (1976). Briefly, infected erythrocytes were cultured in RPAS (RPMI-Albumax-Serum) medium (RPMI 1640 medium (*Gibco*) supplemented with 25 mM HEPES (*Gibco*), 2 mM L-glutamine (*Gibco*), 0.05 mg/ml gentamicin (*Gibco*), 2% human serum and 0.5% of Albumax). Cultures were maintained at 5% hematocrit in a gas mixture of 2% O_2_, 5.5% CO_2_ and 92.5% N_2_ and incubated at 37°C. HbAA and HbAS RBCs from donors were infected with late trophozoite and schizont-infected HbAA erythrocytes obtained after magnetic-activated cell sorting (MACS) (*Miltenyi Biotec*), allowing the separation of erythrocytes infected with hemozoin expressing-parasites. Non-infected blood from the same donors was cultured in the same conditions and the media was changed every day. Infected RBCs were collected after 3 parasite life cycles. A MACS was performed when parasites were mainly late trophozoites and/or early schizonts. After MACS, parasitemia of infected RBCs was adjusted at 45% with non-infected RBCs providing from the same blood donor. Non-infected RBCs were also collected for ghost’s production.

For proteome and phosphoproteome analysis, fresh blood from 3 HbAA and 3 HbAS donors was used. Ghosts and parasite proteins were prepared on three occasions, with one HbAA and one HbAS sample each time (HbAA_1_ and HbAS_1_ were used simultaneously, so as HbAA_2_ with HbAS_2_, and HbAA_3_ with HbAS_3_). As phosphorylation of proteins can change with time after blood collection  (Azouzi et al., 2018), HbAA and HbAS blood were sampled the same day, washed, infected and collected at the same time. The same experimental procedure was performed for all coupled HbAA-HbAS samples.

### Determination of *Plasmodium falciparum* Growth by Flow Cytometry

Growth assays were performed with cryoconserved erythrocytes (from HbAA_1_, HbAA_2_, HbAS_1_ and HbAS_2_ blood donors) that were thawed for this experiment. Parasitemia was determined by flow cytometry every 24hrs. For each condition, 100 µl of resuspended culture were sampled and fixed with phosphate-buffered saline (PBS)-1% paraformaldehyde for 30 minutes at room temperature. Then, 10^11^ cells (in PBS) per well were distributed in a 96-well plate. RBCs were labeled with PBS – Hoechst (BD Biosciences, Hoechst 34580) (HO) (1 µg/ml) and incubated 45 minutes at 37°C in the dark before acquisition and analysis by flow cytometry (FACS Canto II BD). Using FlowJo software, the geomean values of HO fluorescence were used to characterize the parasitemias.

For parasites growth in fresh blood for proteomics analysis, blood smears were also performed every 24hrs and observed by microscopy to follow *P. falciparum* growth and observe the parasite stages.

### Preparation of Erythrocyte Ghosts

HbAA and HbAS erythrocyte ghosts (RBC membranes and cytoskeleton) were obtained by hypotonic lysis at 4°C of either infected or non-infected erythrocytes according to a protocol from Azouzi et al. (2015). The RBCs were first washed three times in PBS 1X and then incubated with 10 volumes of lysis buffer (5 mM Na_2_HPO_4_, 0.35 mM EDTA, pH 8.0 with proteases (*Roche Diagnostics*) and phosphatases inhibitors (*Sigma-Aldrich*)) for 5 minutes on ice. Solutions were afterwards centrifuged at 50,000 x g for 20 minutes at 4°C. For the infected samples, the ghost layer was collected in a new tube to separate ghosts from parasite pellet. The reddish supernatant (containing hemoglobin) was removed, and the ghosts and parasites were washed in lysis buffer until the pellets became colorless. Protein extraction from erythrocyte membranes and parasites was performed by adding one volume of extraction buffer 3X (3% NP40 (*Sigma-Aldrich*), 3% SDS (*Fluka*), 90 mM Tris pH 8, 3 mM EDTA, pH 8 with proteases (*Roche Diagnostics*) and phosphatases inhibitors (*Sigma-Aldrich*)) to two volumes of each pellet. For parasite extracts, a supplementary sonication step was realized to solubilize the proteins. The protein content was measured by BCA (bi-cinchoninic acid) assay (Micro BCA™ Protein Assay Kit, *ThermoFischer Scientific*). The preparations were finally stored at -80°C until analysis.

### Sample Preparation and Mass Spectrometry

**Figure 1:**
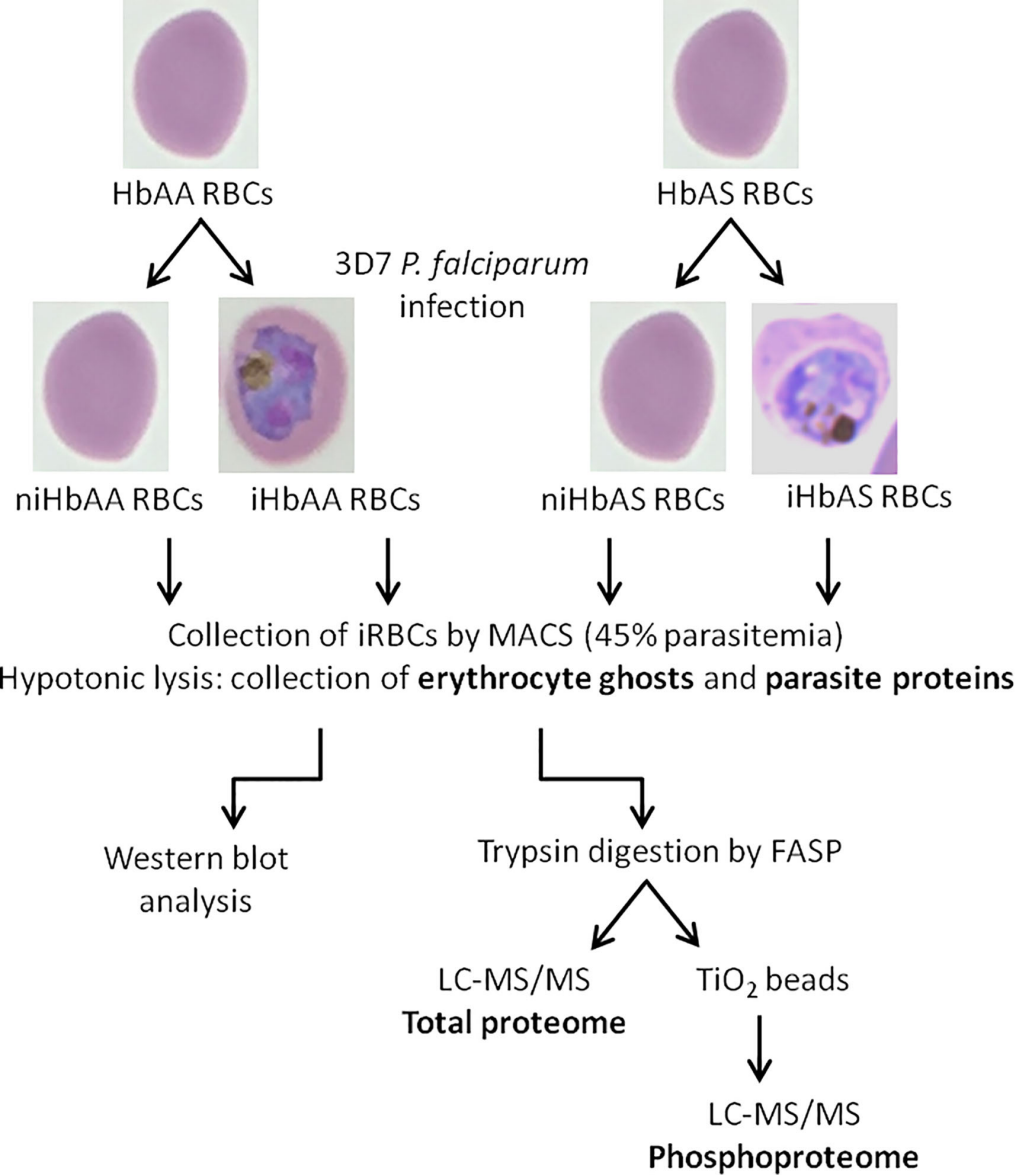
Schematic workflow of infected and non-infected HbAA and HbAS erythrocytes proteome and phosphoproteome experimental procedures – HbAA and HbAS erythrocytes were collected and infected *in vitro* with the 3D7 *P. falciparum* strain. Mature trophozoite/schizont-infected RBCs were collected by MACS (45% parasitemia) and lysed to produce ghosts and collect parasites. Membrane erythrocyte lysates were analyzed by western blot. For mass spectrometry, after trypsin digestion of proteins, phosphopeptides were enriched on TiO_2_ columns and analyzed by LC-MS/MS. Total proteomes of erythrocyte and parasite proteins were also generated. i, infected; ni, non-infected.

Proteins, from erythrocyte membrane or from parasite extracts, (100 μg) were first solubilized in 2% SDS pH 7.5 and heated at 95°C for 5 minutes. Samples were then reduced with 0.1 M dithiothreitol (*Sigma-Aldrich*) at 60°C for 1 hour. Mass spectrometry (MS) sample preparation was performed using a filter aided sample preparation (FASP) method according to Lipecka et al. (2016). We set aside 10% of the peptides for total ghost proteome analysis, while the remaining sample was used for phosphopeptide enrichment, according to the schematic workflow (**Figure 1**).

### Phosphopeptide Enrichment by Titanium Dioxide (TiO_2_)

Phosphopeptide enrichment was carried out using a Titansphere TiO_2_ Spin tips (3 mg/200 μL, Titansphere PHOS-TiO (*GL Sciences Inc*) on the digested proteins for each biological replicate according to manufacturer’s instructions. Samples were applied to the TiO_2_ Spin tips three times in total in order to increase the adsorption of the phosphopeptides to the TiO_2_. Phosphopeptides were eluted by the sequential addition of 50 µL of 5% NH_4_OH and 50 µL of 5% pyrrolidine. Phosphopeptides were washed and eluted with 70 µL of 0.1% trifluoroacetic acid (TFA), 80% acetonitrile high-performance liquid chromatography (HPLC)-grade (1,000 x g for 3 minutes) and vacuum dried.

### NanoLC-MS/MS Protein Identification and Quantification

Samples were resuspended in 10 µL of 0.1% TFA in HPLC-grade water. For each run, 1 µL was injected in a nanoRSLC-Q Exactive PLUS (RSLC Ultimate 3000) (*Thermo Scientific*). Phosphopeptides were loaded onto a µ-precolumn (Acclaim PepMap 100 C18, cartridge, 300 µm i.d.×5 mm, 5 µm) (*Thermo Scientific*), and were separated on a 50 cm reversed-phase liquid chromatographic column (0.075 mm ID, Acclaim PepMap 100, C18, 2 µm) (*Thermo Scientific*). Phosphopeptides were analyzed using mass spectrometry parameters according to Meijer et al. (2017).

The MS files were processed with the MaxQuant software version 1.5.8.3 and searched with Andromeda search engine against a merged database of human from Swissprot 2011-10-19 and *P. falciparum* from Swissprot and PlasmoDB 8.1 (26047 entries in total). MS files from total ghosts (proteome) and from TiO_2_ enriched peptides (phosphoproteome) were searched using two parameters groups. To search parent mass and fragment ions, we set a mass deviation of 4.5 ppm and 20 ppm respectively. Strict specificity for trypsin/P cleavage was required, allowing up to two missed cleavage sites. Carbamidomethylation (Cysteine) was set as fixed modification, whereas oxidation (Methionine) and N-term acetylation were set as variable modifications. For the analysis of MS files issued of TiO_2_ enrichment, the variable modification of phosphorylation on S, T and Y was also added. The false discovery rates (FDRs) at the protein and peptide level were set to 1%. Scores were calculated in MaxQuant as described previously  (Cox and Mann, 2008). Match between runs was allowed. The reverse hits were removed from MaxQuant output. Proteins were quantified according to the MaxQuant label-free algorithm using label-free quantification (LFQ) intensities  (Cox and Mann, 2008; Cox et al., 2014) and phosphopeptides according to intensity; protein and peptide quantification were obtained using at least one peptide per protein.

Statistical and bioinformatic analysis, including heatmaps, profile plots and clustering, were performed with Perseus software (version 1.6.0.7) freely available at www.perseus-framework.org  (Tyanova et al., 2016). For statistical comparison of the ghost proteome, we set four groups niHbAA, iHbAA, niHbAS, iHbAS. Each group contained three biological replicates (different donors). Each sample was also run in technical triplicates.

For total ghost proteome analysis, we analyzed the proteingroups.txt file. Protein LFQ intensities were transformed in log (2). Proteins were separated into *Plasmodium* and human to select the RBC derived proteins. We filtered the data to keep only proteins with at least three valid values in at least one group. Data were imputed to fill missing data points by creating a Gaussian distribution of random numbers with a standard deviation of 33% relative to the standard deviation of the measured values and 1.8 standard deviation downshift of the mean to simulate the distribution of low signal values. We performed an ANOVA test, *p*-value<0.01, S0 = 0.1.

For phosphopeptide analysis, we analyzed the Phospho(STY).txt file. Phosphopeptide intensities were transformed in log (2), site table was expanded to analyze all phosphosites separately. Phosphosites were separated into *Plasmodium* and human to select the RBC derived phosphoproteins. We filtered the data to keep only phosphosites with at least three valid values in at least one group. Only phosphosites with at least 0.75 of localization probability were retained. Phosphosite intensities were normalized by subtracting the median and adding a constant (25). Data were imputed to fill missing data as described above. We performed an ANOVA to compare the four groups, *p*-value<0.05, S0 = 0.1. To evaluate the influence of the genotype on the infection, and if it induces variations, we also performed a two-way ANOVA test, separating the infection and the genotype factors, interaction *p*-value<0.05. Data were imputed to fill missing values points by creating a Gaussian distribution of random numbers with a standard deviation of 33% relative to the standard deviation of the measured values and 3 standard deviation downshift of the mean to simulate the distribution of very low signal values. We performed a *t-*test to compare the parasite phosphoproteome after infection in HbAA vs HbAS, FDR<0.05, S0 = 0.5.

Hierarchical clustering of phosphosites/proteins that survived the tests was performed in Perseus on log-transformed LFQ intensities after z-score normalization of the data, using Euclidean distances.

The mass spectrometry proteomics data have been deposited to the ProteomeXchange Consortium *via* the PRIDE (Perez-Riverol et al., 2019) partner repository with the dataset identifier PXD023280.

### SDS-PAGE and Western Blot

To complete our proteome and phosphoproteome analysis, all erythrocyte ghost samples were also analyzed by anti-Band 3 and anti-pY Band 3 western blots (**Figure 1**).

Ghost lysates (20 µg) were mixed with 5X loading buffer (1.25 mM sucrose, 20% SDS, 250 mM Tris-HCl, 25% β-mercaptoethanol, 1% bromophenol blue, pH 6.8) and heated at 70°C for 10 minutes. Proteins were separated by 4-12% gradient SDS-PAGE under reducing conditions and then transferred to nitrocellulose membrane (Trans-Blot^®^ Turbo™ RTA Mini Nitrocellulose Transfer Kit) (*BioRad*). Blots were blocked with tris buffered saline (TBS) – 5% bovine serum albumin (BSA) (*Roche Diagnostics*), then probed with primary antibodies (mouse anti-Band 3 monoclonal IgG 1,20000 (*Sigma-Aldrich*), rabbit anti-pY^359^ Band 3 polyclonal IgG 1,10000 (*Abcam*), rabbit anti-pY^21^ Band 3 polyclonal IgG 1,10000 (*Abcam*) or rabbit anti-p55, 1,50000, used as a loading control, in TBS- 5% BSA) and finally with the appropriate peroxidase-conjugated secondary antibody (goat anti-rabbit polyclonal IgG 1,5000 (*Jackson ImmunoResearch (Interchim)*), or goat anti-mouse polyclonal IgG 1,5000 (*Jackson ImmunoResearch (Interchim))* applied in TBS - 5% BSA. Detection was performed using the electrochemiluminescence (ECL) Plus Western blotting detection system (*Amersham Biosciences*).

Anti-pY^21^ Band 3/- pY^359^ Band 3 and p55 western blots were performed on the same membrane. These membranes were then stripped and incubated with mouse anti-Band 3 antibody. After ensuring that the loading was similar for each condition with p55 western blots, Image Lab 6.0.1 software was used to measure the intensity of pY^21^ Band 3, pY^359^ Band 3 and Band 3 band signals. Subsequently, as inter-individual variations cannot be excluded, pY^21^ Band 3 or pY^359^ Band 3 intensities were normalized according to the corresponding Band 3 intensity for all donors.

## Results

### Characterization of Hemoglobin Genotype of Blood Donors

For all the experiments, we obtained blood from three abnormal Hb carriers (HbAS_1_, HbAS_2_, and HbAS_3_), and from three normal Hb carriers (HbAA_1_, HbAA_2_, HbAA_3_). We verified their Hb genotype by PCR-RFLP and compared their profiles to control DNA. We confirmed that all the abnormal Hb carriers were HbAS and all the normal Hb ones were HbAA (**Supplementary Figure 1**). All information concerning the donors and their genotypes is summarized in **Supplementary Table 1**.

### Parasite Growth Is Slower in HbS Heterozygous Carriage Than in HbAA Red Blood Cells

**Figure 2:**
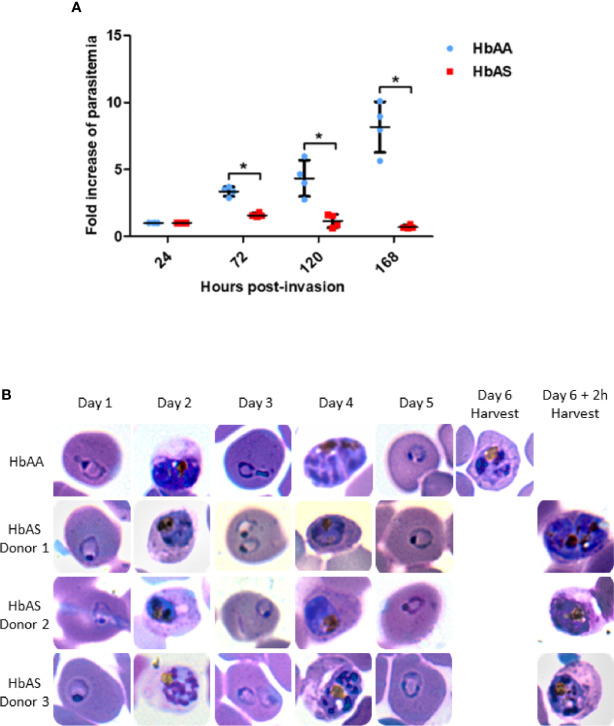
Parasite replication in HbAA and HbAS RBCs assessed by flow cytometry **(A)** and by blood smears **(B)**. Invasion of 2 HbAA (HbAA1 and HbAA2) and 2 HbAS (HbAS1 and HbAS2) blood samples from the cryobank of the the French National Immunohematology Reference Laboratory (CNRGS), was realized at t = 0h. Parasitemia was assessed every 24h. To visualize the fold increase of parasitemia, all parasitemia values were divided by the initial parasitemia measured 24h post-infection. Technical duplicates were performed for each donor, and replicate values for each point were loaded on the graph. Unpaired Mann-Whitney t-test (p-value<0.05) was used to compare values from HbAA and HbAS groups at 24h, 72h, 120h and 168 h., asterisks (*) indicate significantly different values **(A)**. Parasite development in one HbAA (HbAA1 as a reference) and 3 HbAS (HbAS1, HbAS2 and HbAS3) blood donors erythrocytes. Invasion of fresh blood samples was realized at t = 0h. Blood smears were performed every 24h for six days. At day 6, blood smears were realized right before MACS collection **(B)**.

Before collecting infected erythrocytes in order to prepare ghosts and harvest parasites, we compared *P. falciparum* growth in HbAA versus HbAS RBCs. We assessed parasite replication rates by flow cytometry in RBCs provided from two HbAA and two HbAS donors. We observed a significant slower replication rate for *P. falciparum* grown in HbAS RBCs (**Figure 2**). Although parasite growth seems very low for parasites inside HbAS erythrocyte, parasites grew, and progressed from schizonts to ring and from ring to schizonts in HbAS erythrocytes. Microscopic observation revealed no difference in the proportions of different parasites developmental stages during the two first cycles of replication up to 96 h post-infection. From the third cycle onwards, however, we observed a slight retardation of parasite growth in HbAS erythrocytes (**Figure 2B**).

### Erythrocyte Membrane Proteome as a Function of *Plasmodium falciparum* Infection and/or Abnormal Hemoglobin S Carriage

Therefore, to investigate the proteome and phosphoproteome of *Plasmodium falciparum*-infected HbAA and HbAS erythrocytes, parasite development in the two types of RBCs was allowed to proceed for three cycles, and was assessed *via* sequential blood smears, to allow for adequate adaptation to the host cells. Infected RBCs were collected at the same parasite stage after 140 ± 2 h post-infection (**Figure 1**). We ensured that parasites were at the same stages (late trophozoites and early schizonts) in HbAA and HbAS RBCs, with blood smears made just prior to MACS (**Figure 2B**).

The total ghost proteome analyses led to identification of 1438 proteins across all samples, of which 910 were human (65%) (**Supplementary Table 2**) and 528 parasite proteins (35%). Most proteins identified were part of the erythrocyte cytoskeleton or transporters, confirming the enrichment in erythrocyte ghosts.

**Figure 3:**
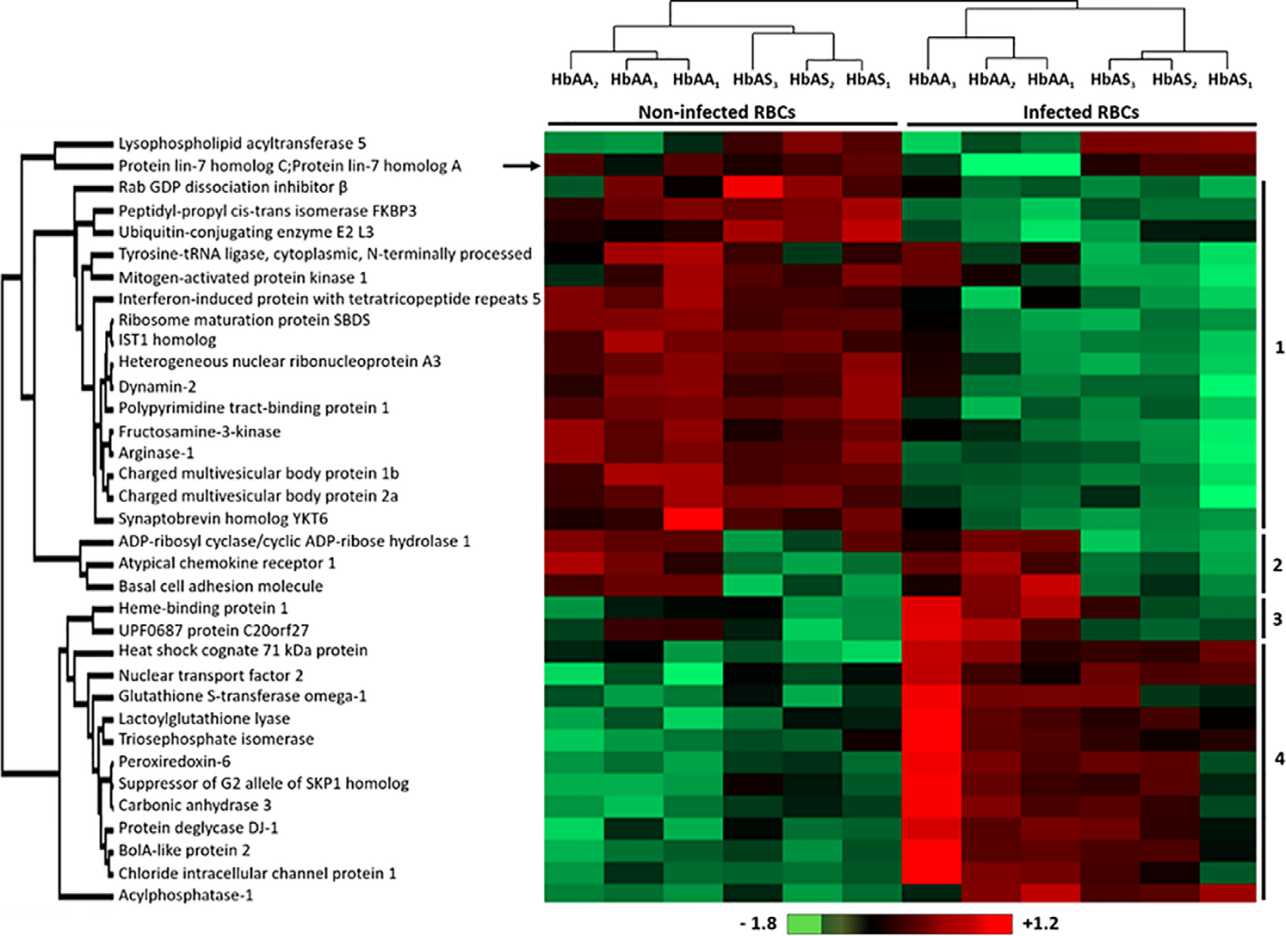
Differentially detected human erythrocyte proteins as a function of *P.* *falciparum* infection and/or HbAS genotype. Six HbAA (non-infected and infected HbAA_1_, HbAA_2_, and HbAA_3_) and six HbAS (non-infected and infected HbAS_1_, HbAS_2_, and HbAS_3_) erythrocyte ghost samples were analyzed by hierarchical clustering based on the detected quantity of RBC proteins (ANOVA test, *p*-value < 0.05). Samples are displayed horizontally (columns) and proteins are shown vertically (rows). The more the protein is represented in light red, the more it is detected in the corresponding sample, and the more it is colored in light green, the less it is detected. The color bar represents log_2_ Z score fold-change. Proteins are clustered according to their quantity profile, represented by dendograms. Black arrow: protein profile that cannot be associated with a cluster.

We observed significant differences in the quantity of 35 erythrocyte proteins according to infection and/or HbS carriage (**Supplementary Table 2**, in green) (*p*-value <0.05) after an ANOVA test. Performing hierarchical biclustering, we identified 4 main clusters of proteins according to their intensity variations (**Figure 3**). Some proteins were of particular interest because their quantity varied according to both *P. falciparum* infection and Hb genotype. Compared to infected RBC and heterozygous HbS carriage, i) the protein lin-7 homolog A/C (black arrow) was detected less frequently in infected HbAA ghosts and ii) Heme-binding protein 1, and UPF0687 protein C20orf27 (Cluster 3), were detected more frequently in HbAA infected samples.

### Erythrocyte Membrane Phosphoproteome as a Function of *Plasmodium falciparum* Infection and/or Abnormal Hemoglobin S Carriage

**Figure 4:**
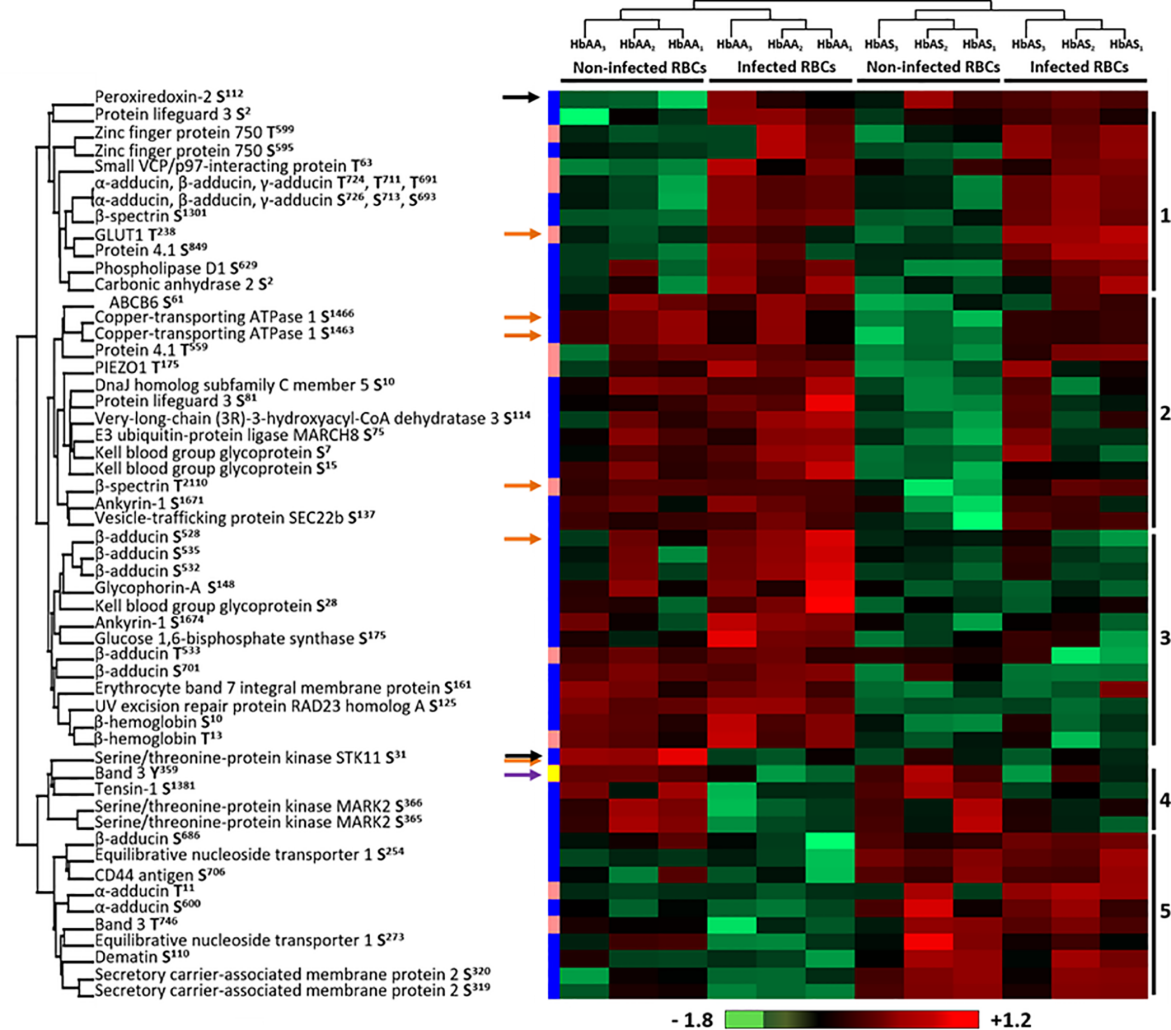
Differentially phosphorylated sites of human erythrocyte proteins as a function of *P.* *falciparum* infection and/or HbAS genotype. Six HbAA (non-infected and infected HbAA_1_, HbAA_2_, and HbAA_3_) and six HbAS (non-infected and infected HbAS_1_, HbAS_2_ and HbAS_3_) were analyzed by hierarchical clustering based on the phosphorylation rate of a specific site of RBC proteins (ANOVA test, *p*-value < 0.05). Samples are displayed horizontally (columns) and proteins are shown vertically (rows). Sites are specified next to the protein name. The more the site is represented in light red, the more it is phosphorylated in the corresponding sample, and the more it is colored in light green, the less it is phosphorylated. The color bar represents log_2_ Z score fold-change. On the left, threonine (T) residues are in pink, serines (S) in blue and tyrosine (Y) in yellow. Proteins are clustered according to their phosphorylation profile, represented by dendograms. Black arrows: protein profiles that cannot be associated with a cluster, orange arrows: phosphosites differentially phosphorylated according to both *P. falciparum* infection and abnormal heterozygous HbS carriage.

Phosphoproteomic analysis led to the quantification of 499 phosphosites from erythrocytic proteins, of which 413 serines (Ser or S), 81 threonines (Thr or T) and only 4 tyrosines (Tyr or Y). Performing an ANOVA test, we observed significant differences in the phosphorylation intensity of several erythrocyte proteins in association with *P. falciparum* infection and/or abnormal HbS carriage (*p*-value<0.05) (**Supplementary Table 3**). We identified 58 differentially phosphorylated sites corresponding to 33 distinct proteins (**Figure 4**). Most of the differential phosphosites were serine residues (44 serines, 13 threonines and 1 tyrosine). Among these phosphosites, 50 had already been described; the 8 remaining were identified in the present work for the first time, corresponding to 6 different new phosphoproteins. For each phosphosite, we ensured that its phosphorylation variation was not due to a different quantity of the corresponding protein, according to the global proteome analysis by LC-MS/MS of all samples (**Supplementary Table 2**).

Hierarchical clustering identified 5 residue phosphorylation modulation profiles (**Figure 4**). Cluster 1 is composed of 11 phosphosites over-phosphorylated in infected erythrocytes, including 5 sites that have already been described as “infection specific” in a meta-analysis of phosphoproteomic studies of *P. falciparum* iRBCs  (Bouyer et al., 2016) (S^726^ and T^724^ of α-adducin, S^1301^ of β-spectrin, T^238^ of GLUT1 and S^849^ of protein 4.1), validating our analytical approach. Cluster 4 included sites presenting decreased phosphorylation intensities due to the infection of RBCs (Y^359^ of Band 3, S^1381^ of Tensin-1, S^365^ and S^366^ of Serine/threonine-protein kinase MARK2). First, these results confirmed previous data by detecting the same phosphorylation sites. Second, these results complete previous data by quantifying the phosphorylation intensities according to Hb genotypes and infection status. The decrease in phosphorylation intensities was not detected in previous studies that analyzed in some cases only infected RBCs. This analysis also identified two clusters containing phosphosites for which the phosphorylation state depended on Hb status, cluster 3 and cluster 5. Pattern 2 and two proteins (black arrows) were of particular interest because they were proteins for which the phosphorylation state was strongly modified depending on the combination of both infection and abnormal Hb carriage. The phosphorylation intensity of S^31^ of Serine/threonine protein kinase STK11 was stronger in non-infected HbAA RBCs. Cluster 2 was composed of S^61^ of ATP-binding cassette transporter sub-family B member 6, S^1463^ and S^1466^ of Copper-transporting ATPase 1, T^559^ of protein 4.1, T^175^ of PIEZO1, S^10^ of DnaJ homolog subfamily C member 5, S^81^ of Protein lifeguard 3, S^114^ of Very-long-chain (3R)-3-hydroxyacyl-CoA dehydratase 3, S^75^ of E3 ubiquitin-protein ligase MARCH8, S^7^ and S^15^ of Kell, T^2110^ of β-spectrin, S^1671^ of Ankyrin-1 and S^137^ of Vesicle-trafficking protein SEC22b. These sites displayed a lower intensity of phosphorylation specifically in non-infected HbAS RBCs. Finally, the phosphorylation intensity of S^112^ of Peroxiredoxin-2 increased in all HbAS samples and infected HbAA ghosts. This protein protects cells against oxidative stress and its function can be regulated by phosphorylation  (Rhee and Kil, 2017). This result may be the consequence of the oxidative stress generated by both *P. falciparum* infection and HbS.

We then performed a two-ways ANOVA to visualize the phosphosites differentially phosphorylated according to both *P. falciparum* infection and abnormal heterozygous HbS carriage (**Supplementary Table 3**) (*p*-value<0.05). Six phosphosites were identified, corresponding to 5 different proteins (orange arrows).

### *Plasmodium falciparum* Infection Modulates Band 3 Tyrosine-Phosphorylation

TiO_2_-based enrichment has similar affinity for the serine, threonine and tyrosine, but the proportion of phosphosites is correlated with the frequency of these phosphosites in the cell. As tyrosine-phosphorylation is less represented, it would require additional steps to collect tyrosine-phosphopeptides or the use of alternative approaches.

**Figure 5:**
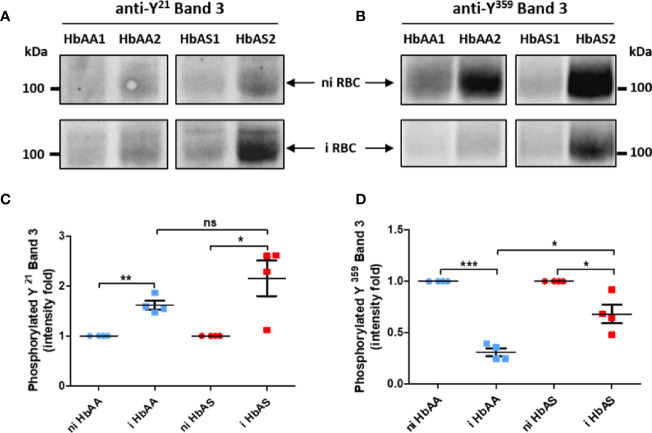
Differences of Band 3 Y^21^ **(A, C)** and Y^359^ **(B, D)** phosphorylation according to P. falciparum infection and abnormal hemoglobin S carriage. 4-12% gradient gels were loaded with 20 µg/lane of ghost protein extracts from 2 HbAA (HbAA1 and HbAA2) and 2 HbAS (HbAS1 and HbAS2) donors. After separation of erythrocyte ghost lysate proteins by SDS-PAGE and transfer on nitrocellulose, tyrosine (Y)-phosphorylation was analyzed using anti-phosphoY21 **(A)** and anti-phosphoY359 **(B)** Band 3 antibodies. Tyrosine phosphorylation intensities were measured with Image Lab software, and these intensities were normalized to Band 3 quantity detected on the same membrane by western blot. For each sample, the phosphorylation intensity value of infected condition was reported to the non-infected condition intensity value **(C, D)**. Independent western blots were realized twice, and technical replicate values for each point were loaded on the graph. Phosphorylation fold intensities for HbAA and HbAS samples were represented in blue and red respectively, with non-infected (circles) and infected (square) ghosts. Paired t-test (*p-value < 0.05; **p-value < 0.01, and ***p-value < 0.001) were performed to compare intensities from the same genotype group (HbAA or HbAS) donors. Unpaired Mann-Whitney t-test (*p-value < 0.05; **p-value < 0.01, and ***p-value < 0.001) was used to compare intensities from infected HbAA and infected HbAS donors. i, infected; ni, non-infected; ns, not significant.

For this reason, the erythrocyte ghosts were also investigated by anti-phospho-tyrosine western blot (**Figure 1**). Band 3 protein is the major known target of erythrocyte tyrosine kinases  (Ferru et al., 2011), and the enhancement of its tyrosine-phosphorylation due to oxidative stress has been described in previous reports  (Pantaleo et al., 2010b; Ferru et al., 2011). Band 3 is known to be phosphorylated upon oxidative stress on several tyrosine residues including Y^21^ and Y^359^ by Syk and Lyn kinases, respectively. We confirmed the modulation of Band 3 Y^359^ phosphorylation in our mass spectrometry analysis (purple arrow in **Figure 4**). Thus, after ensuring that the same amount of protein was loaded for each sample using an anti-p55 western blot, we used specific anti-pY^21^ and anti-pY^359^ Band 3 antibodies (complete Western blots are provided in **Supplementary Figures 2** and **3**). Band 3 Y^21^ phosphorylation intensity increased with *P. falciparum* infection in both HbAA and HbAS erythrocytes (**Figures 5A, C**). We observed that Band 3 Y^359^ phosphorylation intensity decreased with infection in both HbAA and HbAS RBCs (**Figures 5B, D**). This result confirms the profile observed for the same phosphosite in mass spectrometry (**Figure 4**). Furthermore, the decreased phosphorylation intensity of Band 3 Y^359^ is significantly less marked in HbAS than in HbAA RBCs.

### Parasite Phosphoproteome as a Function of Abnormal Hemoglobin S Carriage

**Figure 6:**
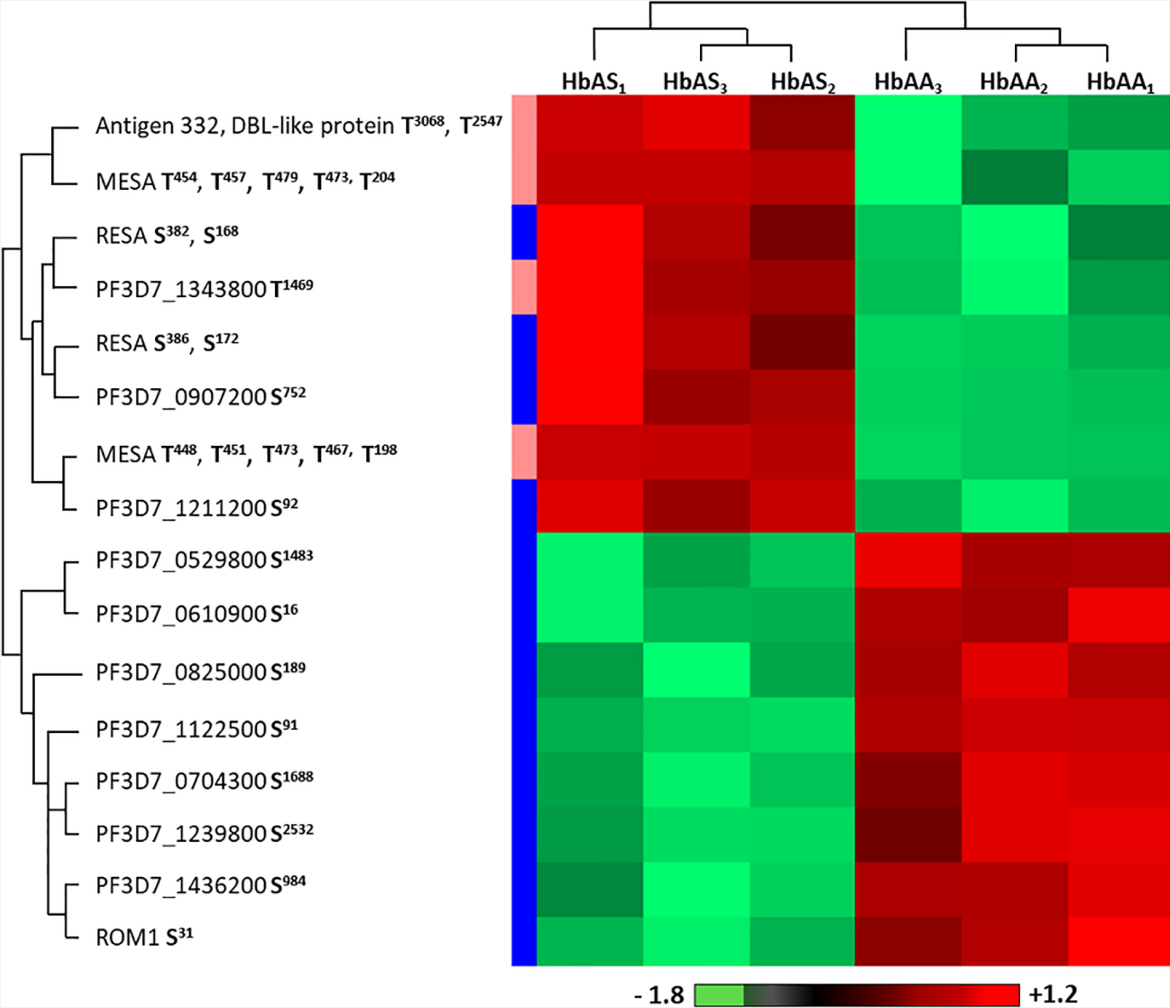
Differentially phosphorylated sites of parasite proteins according to hemoglobin genotype. Parasites from three HbAA (infected HbAA_1_, HbAA_2_, and HbAA_3_) and three HbAS (infected HbAS_1_, HbAS_2_, and HbAS_3_) extracts were analyzed by hierarchical clustering based on the phosphorylation’s rate of a specific site of RBC proteins (Student’s *t*-test, *p*-value < 0.05). Samples are displayed horizontally (columns) and proteins are shown vertically (rows). Phosphosites are specified next to the protein name. The more the site is represented in dark red, the more it is phosphorylated in the corresponding sample, and the more it is colored in light green, the less it is phosphorylated. The color bar represents log_2_ Z score fold-change. On the left, threonine (T) residues are in pink and serines (S) in blue. Proteins are clustered according to their phosphorylation profile, represented by dendograms.

Within the proteome and the phosphoproteome analysis, we also focused on the parasite proteins and we analyzed them according to Hb genotype of the erythrocyte in which *P. falciparum* grew (**Figure 1**). Proteome analysis revealed no significant difference in the protein amounts, with profiles showing individual variability. Phosphoproteome analysis identified 27 phosphosites that were differentially phosphorylated according to Hb genotype, corresponding to 14 different phosphoproteins (Student *t*-test, *p*-value<0.05) (**Supplementary Table 4**). Performing hierarchical clustering, we could identify two patterns of phosphorylation profiles (**Figure 6**). Cluster 1 included phosphorylation sites in 8 proteins such as ring-infected erythrocyte surface antigen (RESA), Antigen 332 – DBL like protein, mature parasite-infected erythrocyte surface antigen (MESA), and unknown proteins with the following PlasmoDB identifiers, MAL13P1.380 (PF3D7_1343800), PFI0345w (PF3D7_0907200), PFL0555c (PF3D7_1211200) for which phosphorylation intensities were greater when parasites were cultured in HbAS RBCs than in HbAA RBCs. Cluster 2 was composed of phosphosites from 8 proteins, including Rhomboid protease 1 (ROM1), PFE1485w (PF3D7_052980), PF07_0016 (PF3D7_0704300), PFL1930w (PF3D7_1239800), PF11_0233 (PF3D7_1122500), MAL8P1.29 (PF3D7_0825000), PF14_0343 (PF3D7_1436200) and PFF0535c (PF3D7_0610900). All the phosphosites of cluster 2 were serines and showed higher phosphorylation intensities in parasites grown in HbAA compare to HbAS erythrocytes.

## Discussion

This project aimed to measure the impact of HbS on proteomic and phosphoproteomic regulation of *P. falciparum-*infected erythrocyte membrane proteins. We considered the impact of oxidative stress generated by both HbS heterozygous carriage and *P. falciparum* infection, because it could disturb the kinase/phosphatase equilibrium. Previous studies have investigated the phosphoproteome of HbSS and normal RBCs  (George et al., 2010), or of HbAA erythrocytes after *P. falciparum* infection  (Bouyer et al., 2016). To our knowledge, however, the phosphoproteome of HbAS RBCs has never been studied before. This study is also the first experimental, comparative and quantitative analysis of parasite and human protein phosphorylation of HbAA- and HbAS-infected erythrocyte membranes.

Other erythrocyte genetic abnormalities, such as α-thalassemia and glucose-6-phosphate dehydrogenase (G6PD) deficiency, can also confer protection against malaria  (Taylor et al., 2013; Kakande et al., 2020) and coexist in populations affected by HbS in malaria endemic areas. Thus, the co-carriages of these other RBC disorders with HbS have been considered in this work (**Supplementary Figures S4–S6**). Investigation in the present study of cumulative erythrocyte disorders along with HbS led to the identification of G6PD deficiency carriages for the HbAS_2_ and HbAS_3_ donors. Also, HbAS_2_ and HbAS_3_ were heterozygous for α-thalassemia. We cannot exclude that these additional mutations may have an effect on *Plasmodium falciparum* infection. It is of note that the consequences of the interactions between sickle cell trait and G6PD deficiency with respect to malaria resistance conferred to carriers is still controversial. Further studies need to be performed to analyze and identify any epistasis between these two genetic disorders  (Esoh and Wonkam, 2021). In our proteomics works, statistical analyses were performed in order to conserve only proteins that were differentially phosphorylated or abundant in all the HbAS samples compared to all HbAA samples. Moreover, our cohort, albeit small, reflects the reality, as the co-carriage of HbS with G6PD deficiency and alpha-thalassemia are frequently found in malaria endemic areas  (Chauvet et al., 2019; Okafor et al., 2019). It is also important to note here that one of our donors, HbAS_1_, only has sickle cell trait. However, to decipher accurately the impact of each mutation and of each combination of mutations on parasite development, further studies with large cohorts including individual carriage of these different genetic disorders, and all possible combinations of co-carriage of genetic disorders, should be conducted. Our own published work highlighted the importance of considering multiple RBC genotypes in studies of parasite antigen presentation at the erythrocyte surface.

*P. falciparum* growth was compared in HbAA and HbAS RBCs in our culture conditions. Indeed, parasite development in HbAS erythrocytes can be inhibited according to culture medium and atmospheric conditions, notably in hypoxia  (Archer et al., 2018). We followed the course of parasite growth either by flow cytometry or blood smears over at least three cycles, in RBCs from HbAA and HbAS donors, and observed a lower replication rate for parasites grown in HbAS RBCs. These data could be seen as consistent with HbAS-conferred resistance against *P. falciparum* malaria. Although it is better to work with fresh blood, previous studies demonstrated similar results with cryoconserved erythrocytes  (Egan et al., 2018; Kariuki et al., 2020), so for convenience, cryoconserved RBCs can be used.

**Figure 7:**
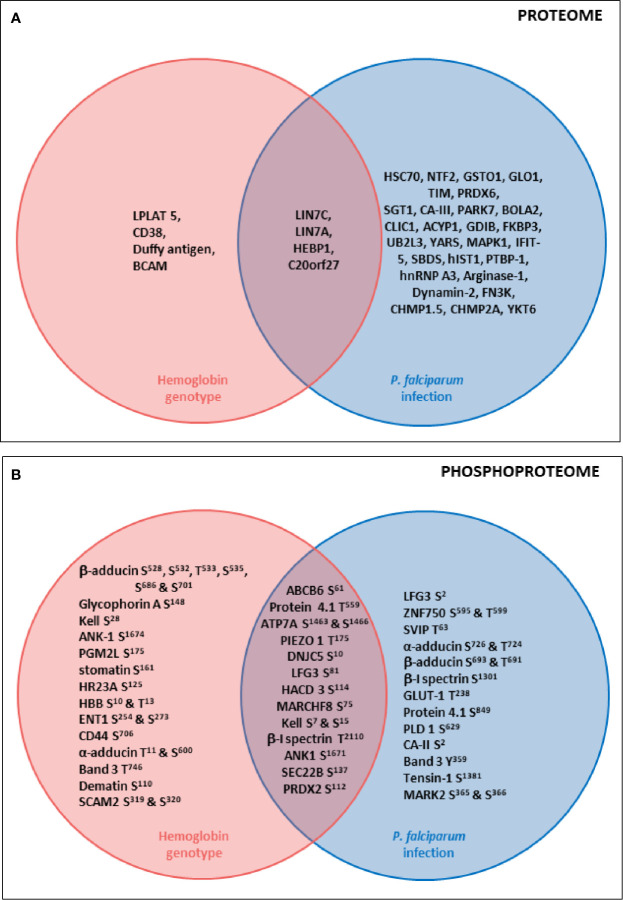
Summary diagrams of proteome **(A)** and phosphoproteome **(B)** modulations of erythrocyte proteins according to *P. falciparum* infection and HbS carriage. Venn diagrams describing proteins whose quantity **(A)** or phosphorylation intensity **(B)** are varying according to hemoglobin genotype, *P. falciparum* infection or both parameters. Uniprot short names of proteins were represented, and phosphosites indicated. Erythrocyte proteins whose quantity **(A)** or phosphorylation intensity **(B)** was varying according to only hemoglobin genotype (ANOVA test) were presented in the pink circle. Erythrocyte proteins whose quantity **(A)** or phosphorylation intensity **(B)** was varying according to only *P. falciparum* infection (ANOVA test) were presented in the blue circle. Erythrocyte proteins whose quantity **(A)** or phosphorylation intensity **(B)** was varying according to both hemoglobin genotype and *P. falciparum* infection (ANOVA test) were presented in the junction between the pink and the blue circle.

Concerning erythrocyte proteins, modifications of both quantity and phosphorylation were identified, as summarized in **Figure 7**. Proteomic analysis revealed a group of proteins for which the quantity varied according to both infection and HbAS genotype. The current literature on Protein Lin-7 homolog A/C in red cells does not allow us to explain the decrease in its quantity in iRBC HbAA. However, the HEBP1 protein was of particular interest. Indeed, this protein binds to free porphyrinogens and facilitates the elimination of elements potentially toxic for the cell  (Jacob Blackmon et al., 2002). To our knowledge, no link between this protein and *P. falciparum* infection metabolism has ever been established. However, one can imagine that this protein could be necessary for the detoxification of the free heme generated during *P. falciparum* growth, because the quantity of this protein detected at the erythrocyte membrane increases with infection in HbAA RBCs. Heme detoxification is a key mechanism for parasite development, and is notably targeted in the development of drugs for malaria. However, the quantity of HEBP1 detected by mass spectrometry analysis does not increase in HbAS ghosts with *P. falciparum* infection. Therefore, it can be hypothesized that hemozoin formation and heme detoxification could be altered in HbAS RBCs.

Furthermore, the « Atypical chemokine receptor 1 » protein, also known as Duffy antigen, was detected at lower levels in the HbAS erythrocyte membrane. The Duffy antigen is known to be a receptor for the parasite *Plasmodium vivax*. Moreover, the Duffy null blood group confers protection against *P. vivax* malaria* * (Langhi and Bordin, 2006). To our knowledge, protection conferred by this blood group against *P. falciparum* malaria has never been described. A recent study reported a clinical case where a HbSS individual did not present any protection against *P. vivax* malaria  (Moiz and Majeed, 2018). However, a more frequent association between HbS and Duffy null blood group has been observed, this combination being potentially selected in areas where *P. falciparum* and *P. vivax* malaria are both frequent  (Gelpi and King, 1976), in particular in sub-Saharan Africa, a region from which the 3 HbAS blood donors originate. But for now, these observations don’t allow us to suggest any mechanistic hypothesis regarding the potential role of a lower amount of Duffy antigen in conferring resistance to *P. falciparum* RBC invasion in HbAS carriers.

In our phosphoproteome analysis, proteins from 4 main groups were identified : (i) proteins from the Ankyrin-R complex (ankyrin, Band 3, β spectrin, stomatin, and glycophorin A); (ii) proteins from the junctional complex (protein 4.1, dematin, α, β and γ adducins, CD44, Duffy and Kell); (iii) carriers and channels (GLUT1, ENT1, PIEZO1, Secretory carrier-associated membrane protein 2, ABCB6 and Band 3); and (iv) kinases (Serine/threonine-protein kinase MARK2, Serine/threonine-protein kinase STK11). These phosphorylation changes could have an impact on the function of the different proteins, and thus on the structure and stability of the membrane of iRBCs. Indeed, phosphorylation of β spectrin by the casein kinase 1, associated with the membrane, leads to decreased mechanical stability of the membrane  (Manno et al., 1995). Phosphorylation of dematin by the parasite kinase FIKK  (Brandt and Bailey, 2013), or by a PKA, seems to disturb its function, interfering with its binding with actin and spectrin  (Lalle et al., 2011). Finally, phosphorylation of protein 4.1 following infection  (Chishti et al., 1994) inhibits its ability to promote the interaction between actin and spectrin, reducing membrane stability  (Manno et al., 1995) and could participate in parasite-induced modifications of membrane properties.

These modifications could also modulate erythrocyte membrane permeability. During its intra-erythrocytic development, *P. falciparum* uses some of these transporters for its metabolism. In our work, we have shown that the protein ABCB6, a heme transporter, displayed lower phosphorylation intensity in non-infected HbAS RBCs, compared to infected HbAS, and infected or non-infected HbAA erythrocytes. However, a study recently observed that ABCB6-deficient RBCs were resistant to invasion by *P. falciparum * (Egan et al., 2018).

The two-way ANOVA test highlighted sites at which the phosphorylation intensity varied according to both infection and HbAS genotype. The lack of antibodies corresponding to those phosphosites did not allow us to validate the observed variations of phosphorylation intensities by western blot. All the identified modifications could thus be involved in regulation of *P. falciparum* development and in molecular interactions in the membrane protein complexes, such as the cytoadherence complex, which is associated with the cytoadherence of iRBCs and therefore with severe forms of malaria.

Concerning parasite proteins, although the proteome appeared unchanged according to our observations, the phosphorylation intensities of serines and threonines of some parasite proteins, such as RESA, MESA, *Pf*332 and ROM1, did vary according to the Hb genotype of the RBCs. RESA is a protein involved in parasite RBC invasion that is expressed from the ring stage, where it plays a major role in the reduction of membrane deformability. After invasion, RESA is phosphorylated and binds close to the site of auto-association of β spectrin dimers. This binding stabilizes the spectrin tetramer, by preventing the dimer dissociation that is necessary for invasion, strengthening the RBC membrane and preventing other invasion events  (Pei et al., 2007). MESA is a phosphoprotein associated with the erythrocyte skeleton that interacts with 4.1 protein, at a position involved in the formation of the ternary complex (between p55, GPC and 4.1). MESA competes with p55 for the binding of 4.1 protein, and could thus impact iRBC membrane skeleton stability  (Waller et al., 2003). Binding of MESA with 4.1 protein is crucial for parasite survival in the RBC, and also for correct localization of MESA in the erythrocyte skeleton  (Black et al., 2008). MESA also binds to the cytoskeletal protein ankyrin (Kilili et al., 2019). Moreover, *Pf*332, a MCs resident peripheral protein, is associated with the erythrocyte cytoskeleton, at the schizont stage, where it binds to actin  (Waller et al., 2010). *Pf*332 plays a role in membrane rigidity reduction, for *Pf*EMP1 trafficking and for iGRs adherence  (Glenister et al., 2009). Indeed, RBCs infected by *Pf*332-deficient parasites are more rigid, express less *Pf*EMP1 and display lower adherence to CD36. Finally, ROM1 plays a role in the parasitophorous vacuole formation  (Vera et al., 2011). It is also able to cleave AMA1, and adhesins during parasite invasion. There is no evidence in the literature of parasite-protein sites for which modifications of phosphorylation intensity have been described. However, the corresponding proteins play a role in iRBC membrane and cytoskeleton remodeling, in order to modify their rigidity and adherence properties. Thus, we can hypothesize that the altered cytoadherence and remodeling of HbAS iRBCs membranes could be linked to the alteration of the phosphorylation status of these proteins. Moreover, our results suggest that HbS is also able to modulate parasite functions. One could also hypothesize that the parasite is able to adapt itself to the different environment of these abnormal RBCs by influencing the phosphorylation balance.

The parasite does not express any classical tyrosine kinase, but only tyrosine kinase-like  (Abdi et al., 2013). Numerous studies have demonstrated the importance of the equilibrium in the tyrosine kinase and phosphatase activities during the intra-erythrocytic parasite life. Band 3 was chosen to study the potential Y phosphorylation differences due to HbAS genotype and infection. An increase of Band 3 Y^21^ phosphorylation intensity with infection was observed for HbAA and HbAS donors. However, a decrease of Band 3 Y^359^ phosphorylation intensity with infection was seen for these same donors. The number of donors is low, and we could not exclude individual variation. However, although not statistically detected in MS, the decreased Band 3 Y^359^ phosphorylation intensity appeared to be less important for HbAS donors than for HbAA donors in our western blot analysis. That the reduction of phosphorylation intensity in HbAS was less than that seen in HbAA iRBCs at the end of the parasite cycle could regulate Band 3 interactions with other erythrocyte membrane proteins and participate in the deregulation of erythrocyte membrane stability. Indeed, Y^359^ belongs to the Band 3 binding domain for 4.1 protein  (Lux, 2016). Moreover, a recent study suggests that regulation of Band 3 phosphorylation is associated with the erythrocyte membrane destabilization needed for merozoite egress at the end of the intra-erythrocytic cycle  (Pantaleo et al., 2017). Variations of Band 3 phosphorylation could be thus associated with altered parasite egress, and could explain the lower parasite replication rate observed in HbAS iRBCs.

Finally, this investigation of the proteome and the phosphoproteome of red cell membrane extracts from *P. falciparum* infected and non-infected erythrocytes, according to HbS heterozygous carriage, allowed us to point out a number of erythrocyte membrane transporters, skeletal proteins and parasite proteins for which phosphorylation was impacted by this genetic abnormality, combined with infection. This study, at the same time novel but also complicated by the use of freshly-collected RBCs, contributes to a better understanding of the biochemical mechanisms involved in conferring protection of HbAS carriers against disease caused by *P. falciparum*.

## Data Availability Statement

The datasets presented in this study can be found in online repositories. The name of the repository/repositories and accession number can be found below: ProteomeXchange *via* the PRIDE database, https://www.ebi.ac.uk/pride/, PXD023280.

## Ethics Statement

The studies involving human participants were reviewed and approved by Necker‐Enfants‐Malades Hospital, Committee for the Protection of Persons n°DC 2014-2272. The patients/participants provided their written informed consent to participate in this study.

## Author Contributions

SA, IG, AL, AM, and FM-N conceived and designed the project. J-AR and SM collected the blood samples. MC, SD, CD, ML, AM, and MO performed the biochemical experiments. MC, AM, FM-N, and DP performed the genotyping. CC, IG, and JL performed the mass spectrometry experiments and analyses. SA, MC, IG, CLVK, AL, AM, and FM-N wrote the paper. All authors reviewed the manuscript before submission. All authors contributed to the article and approved the submitted version.

## Funding

We thank the University of Paris for the doctoral scholarship awarded to MC. Funding came partly from the “Laboratoire d’Excellence GR-Ex,” Paris, France, reference ANR-11-LABX-0051 that is funded by the program “Investissements d’avenir” of the French National Research Agency, reference ANR-11-IDEX-0005-02.

## Conflict of Interest

The authors declare that the research was conducted in the absence of any commercial or financial relationships that could be construed as a potential conflict of interest.

## Abbreviations

BCA, Bi-cinchoninic acid; BSA, Bovine serum albumin; CPDA, Citrate-phosphate-dextrose with adenine; ECL, Electrochemiluminescence; FASP, Filter aided sample preparation; FDR, False discovery rate; G6PD, Glucose-6-phosphate dehydrogenase; Hb, Hemoglobin; HO, Hoechst; HPLC, High-performance liquid chromatography; iRBCs, infected RBCs; LFQ, Label-free quantification; MACS, Magnetic-activated cell sorting; MCs, Maurer’s clefts; MESA, Mature parasite-infected erythrocyte surface antigen; MS, Mass spectrometry; PCR-RFLP, Polymerase chain reaction – restriction fragments length polymorphisms; *Pf*EMP1, *Plasmodium falciparum* erythrocyte membrane protein 1; RBCs, Red blood cells; RESA, Ring-infected erythrocyte surface antigen; ROM1, Rhomboid protease 1; Ser or S, Serine; TBS, Tris buffered saline; TFA, Trifluoroacetic acid; Thr or T, Threonine; Tyr or Y, Tyrosine.
