# Supplementary material for: Sickle Cell Trait Modulates the Proteome and Phosphoproteome of Plasmodium falciparum-Infected Erythrocytes
Source: Front Cell Infect Microbiol. 2021 Mar 24;11:637604. doi: 10.3389/fcimb.2021.637604 (PMC8024585; doi:10.3389/fcimb.2021.637604)
Supplement: Supplementary file 1 [file DataSheet_1.docx]

Supplementary Data

**Supplementary materials and methods**

*Molecular determination of G6PD deficiency and α-thalassemia 3.7 deletion*

G6PD deficiency is an enzymopathy caused by mutations in the *G6PD* gene located on X chromosom. The G6PD B enzyme proteoform ensures normal G6PD activity. The A376G mutation is associated with the G6PD A variant, with a minimal loss in enzyme activity (Carter et al., 2011). The G6PD A^-^ variant, associated with enzyme deficiency, is due to an additional mutation (mainly G202A or T968C in sub-Saharan populations). Alpha-thalassemia is an hemoglobinopathy caused by an α-globin synthesis defect. In sub-Saharan Africa, the most common gene defect encountered is a deletion of 3.7 Kb (α^-3.7^ deletion) (de Medeiros Alcoforado et al., 2012).

G6PD A and A^-^ variants were sought using PCR-RFLP, with a method adapted from Carter *et al.*(Carter et al., 2011), and according to the GoTaqFlexi DNA Polymerase (*Promega*) requirements. When G6PD A variant was identified, the additional mutations G6PD-G202A and G6PD-T968C were then investigated.

Alpha-thalassemia gene deletion was detected by multiplex PCR with a method adapted from Liu *et al.*(Liu et al., 2000), and according to the GoTaqFlexi DNA Polymerase (*Promega*) requirements.

**Supplementary figures**

**Supplementary Figure S1. Determination of Hb genotype profiles by PCR-RFLP.** The DNA fragments of the β-globin gene, containing the mutation site HbS were amplified by PCR, digested by *Dde* I and *Bse* RI restriction enzymes and visualized on 2% agarose gel. The donor’s profiles were compared to those of control DNA to determine the corresponding genotype.


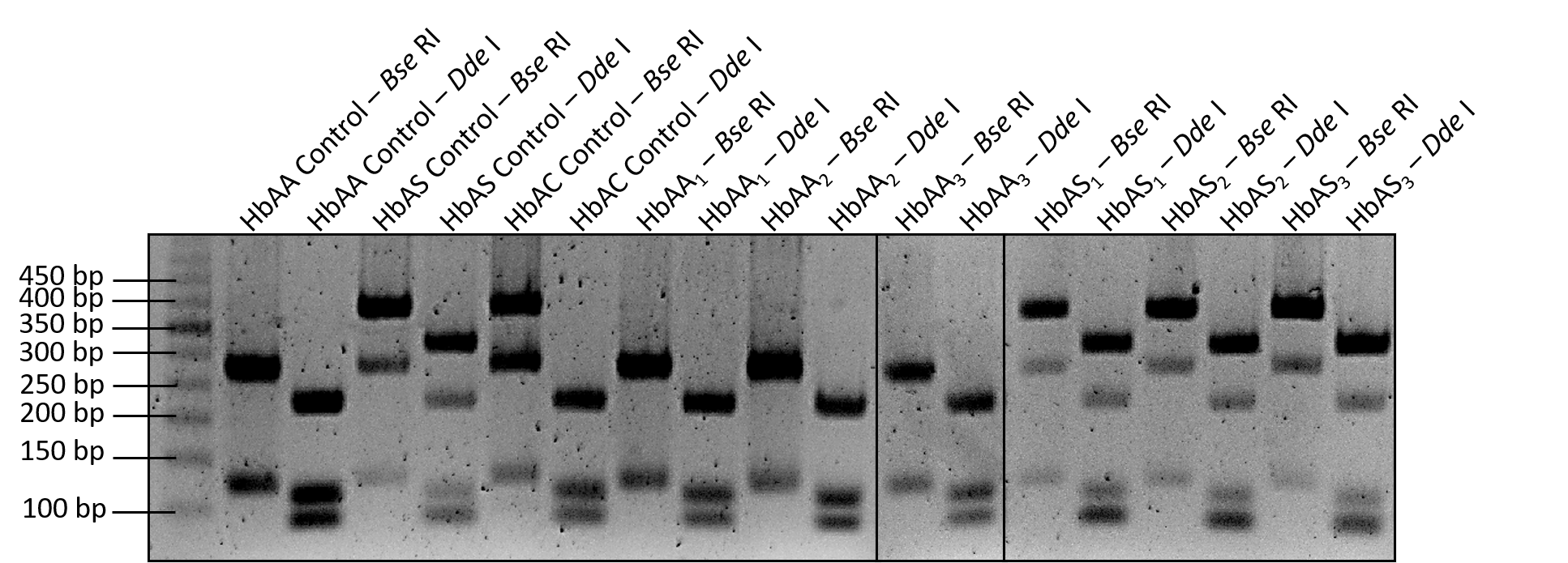


**Supplementary Figure S2. Western blots of Band 3 Y^21^, Band 3 and p55.** 4-12% gradient gels were loaded with 20 µg/lane of ghost protein extracts from 2 HbAA (HbAA_1_ and HbAA_2_) and 2 HbAS (HbAS_1_ and HbAS_2_) donors. After separation of erythrocyte ghost lysate proteins by SDS-PAGE and transfer on nitrocellulose, anti-p55 and anti-phosphoY^21^ Band 3 western blots were performed. Anti-phosphoY^21^ Band 3 western blots were then stripped and incubated with anti-Band 3 antibody.

i: infected. ni: non-infected.


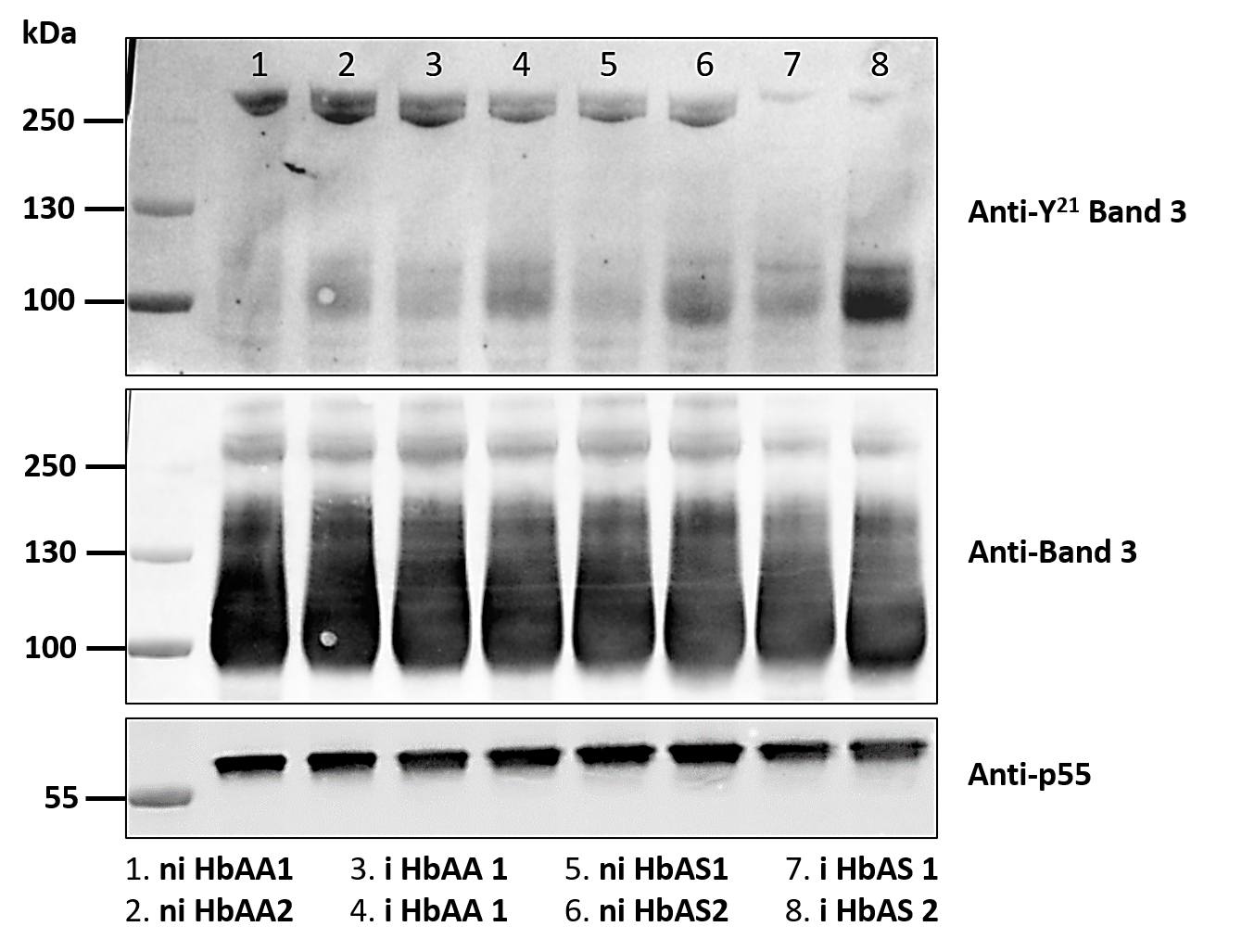


**Supplementary Figure S3.** **Western blots of Band 3 Y^359^, Band 3 and p55.** 4-12% gradient gels were loaded with 20 µg/lane of ghost protein extracts from 2 HbAA (HbAA_1_ and HbAA_2_) and 2 HbAS (HbAS_1_ and HbAS_2_) donors. After separation of erythrocyte ghost lysate proteins by SDS-PAGE and transfer on nitrocellulose, anti-p55 and anti-phosphoY^359^ Band 3 western blots were performed. Anti-phosphoY^359^ Band 3 western blots were then stripped and incubated with anti-Band 3 antibody.

i: infected. ni: non-infected.


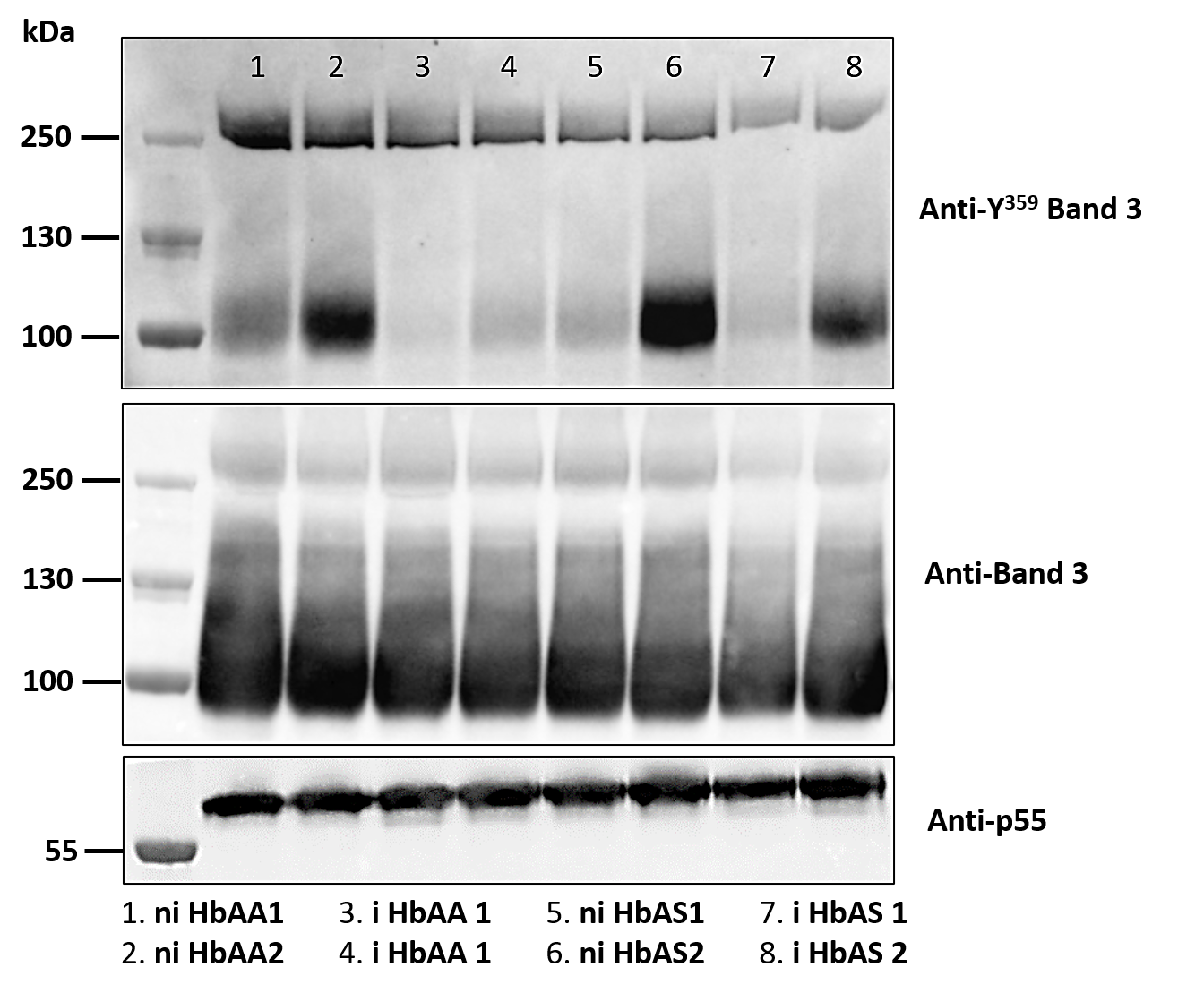


**Supplementary** **Figure S4. Determination of G6PD variant A carriage by PCR-RFLP.** The fragments containing the mutation site G6PD A376T were amplified by PCR, then digested by the restriction enzyme *Fok* I and visualized on 2% agarose gel. The donor’s profiles were compared to those of control DNA to determine the genotype of donors’ samples. All HbAA donors and HbAS_1_ were G6PD BB or B. The donor HbAS_2_ was G6PD AA and the donor HbAS_3_ was G6PD AB.


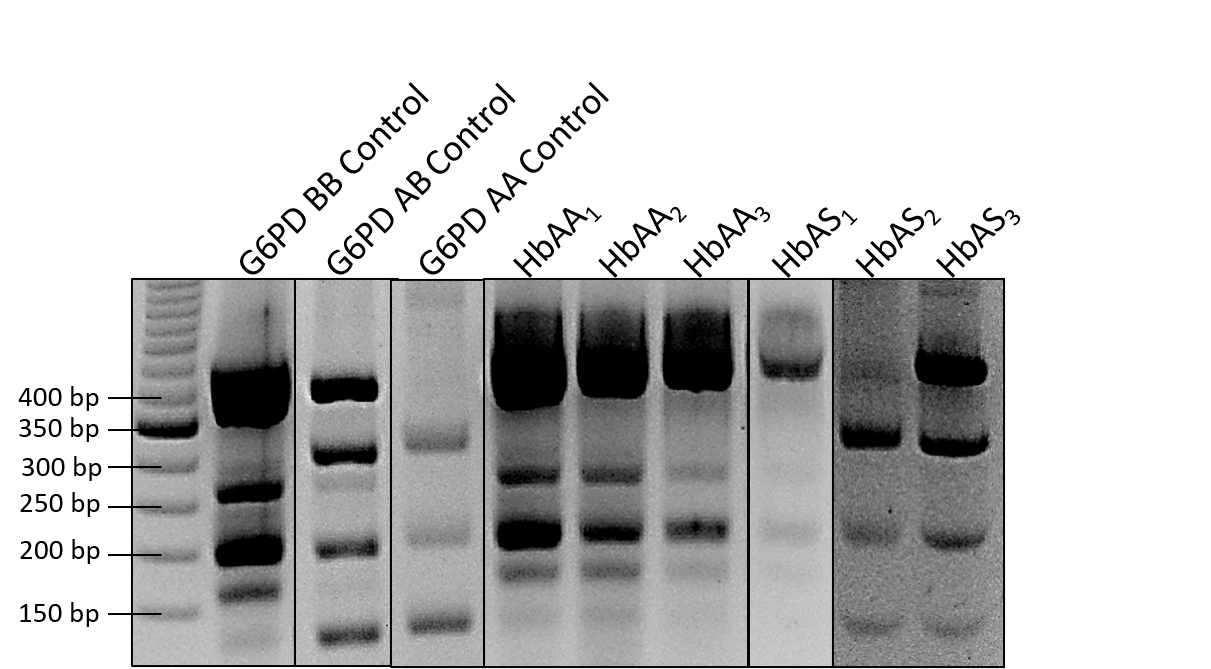


**Supplementary Figure S5.** **Determination of G6PD variant A and A^-^ carriage by DNA sequencing (example for HbAS_2_ Donor).** The fragments containing the mutation site G6PD A376G, G202A and T968C were amplified by PCR, then analyzed by DNA sequencing. The donor HbAS_2_ was G6PD A^-^A^-^ and the donor HbAS_3_ was G6PD AB.


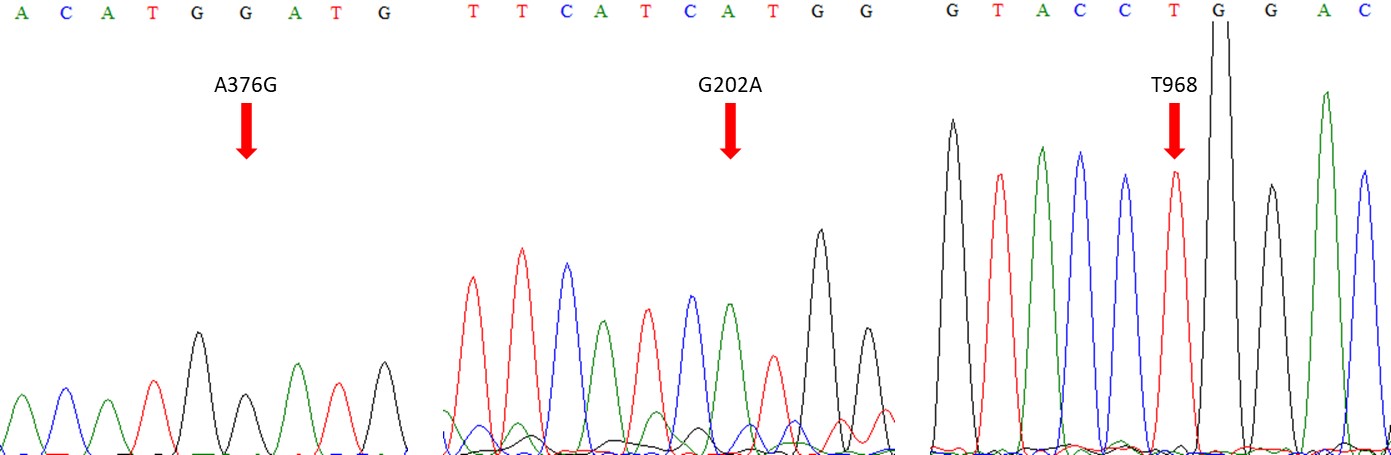


**Supplementary** **Figure S6. Determination of α-thalassemia genotype profiles by multiplex PCR.** The fragments containing the potential deleted sequence were amplified by multiplex PCR and visualized on 1.5% agarose gel. Primers permit to amplify a fragment of 2.1 kb or 1.9 kb if the donor has a deletion in his *α-thalassemia* gene. All donors were αα/αα, except the donors HbAS_2_ and HbAS_3_ who were αα/α^-3.7^.


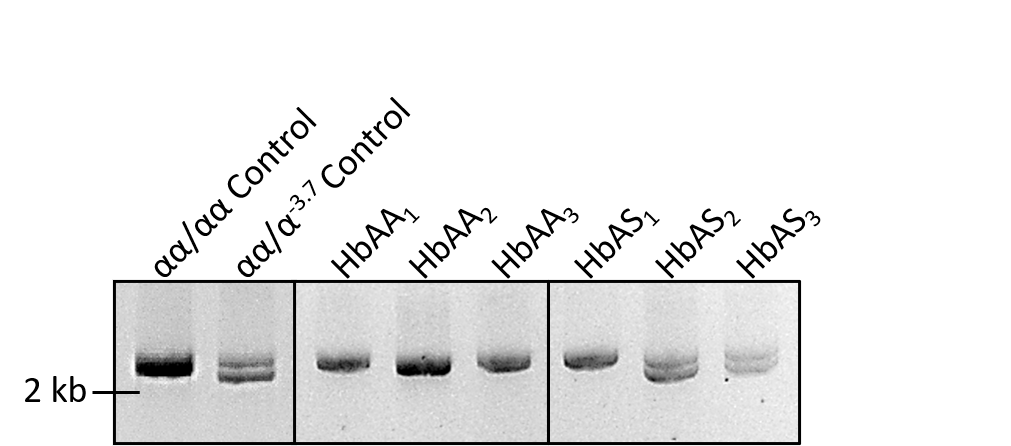


**Supplementary Table 1. Genotype informations of HbAA and HbAS blood donors.** Alpha-thalassemia and G6PD deficiency genotypes were determined for each blood donor and summarized in this table.

| **Blood donor** | **Gender** | **α-thalassemia genotype** | **G6PD genotype** |
| --- | --- | --- | --- |
| **HbAA_1_** | female | αα/αα | G6PD BB |
| **HbAA_2_** | male | αα/αα | G6PD B |
| **HbAA_3_** | female | αα/αα | G6PD BB |
| **HbAS_1_** | male | αα/αα | G6PD B |
| **HbAS_2_** | female | αα/α^-3.7^ | G6PD A**^-^**A**^-^** |
| **HbAS_3_** | female | αα/α^-3.7^ | G6PD AB |

**Supplementary Table 2. Detected human erythrocyte proteins as a function of *P. falciparum* infection and/or HbAS genotype** - 910 human proteins were detected in RBC ghost. Significative (ANOVA) variations of protein amount are highlighted in green (Figure 3).

**Supplementary Table 3. Differentially phosphorylated sites of human erythrocyte proteins as a function of *P. falciparum* infection and/or HbAS genotype** – 499 phosphosites from human proteins were quantified in RBC ghost. Significative (one and two-way ANOVA analysis) variations of protein phosphorylation intensity are highlighted in green (Figures 4 and 5).

**Supplementary Table 4. Differentially phosphorylated sites of parasite proteins according to hemoglobin genotype.** 27 phosphosites from parasite proteins were quantified in iRBC ghost. Significative (Student's *t*-test analysis) variations of protein phosphorylation intensity are highlighted in green (Figure 6).
